# Endoscopic necrosectomy of infected pancreatic Walled-Off Necrosis using two types of Lumen-Apposing Metal Stents

**DOI:** 10.1371/journal.pone.0361859

**Published:** 2026-11-03

**Authors:** Cong Ding, Hui Wang, Yifeng Zhou, Jing Yang, Xia Wang, Goufan Shen, Xiaofeng Zhang, Richard Hu, Jianfeng Yang

**Affiliations:** 1 Department of Gastroenterology, Affiliated Hangzhou First People’s Hospital, School of Medicine, Westlake University, Hangzhou, Zhejiang, China; 2 Key Laboratory of Integrated Traditional Chinese and Western Medicine for Biliary and Pancreatic Diseases of Zhejiang Province, Hangzhou, Zhejiang, China; 3 Hangzhou Institute of Digestive Diseases, Hangzhou, Zhejiang, China; 4 Zhejiang Provincial Key Laboratory of Clinical Cancer Pharmacology and Toxicology Research, Hangzhou, Zhejiang, China; 5 Department of Gastroenterology, Olive View-UCLA Medical Center and David Geffen School of Medicine at UCLA, Los Angeles, California, United States of America; Al-Azhar University, EGYPT

## Abstract

**Background:**

Lumen-apposing metal stent (LAMS) placement enables transmural drainage of walled-off necrosis (WON) with secure anchoring and provides endoscopic access for subsequent necrosectomy. This study compared the efficacy and safety of two commercially available LAMS designs (Hot-Axios vs. Micro-Tech) in patients with infected WON.

**Methods:**

We retrospectively reviewed patients with infected WON who underwent endoscopic ultrasound (EUS)-guided LAMS placement followed by endoscopic debridement at our center between August 2020 and June 2025.

**Results:**

Thirty-one patients were included (Hot-Axios, n = 21; Micro-Tech, n = 10). The overall necrosectomy success rate was 80.65% (25/31). Necrosectomy success was numerically higher with Hot-Axios than with Micro-Tech (85.71% vs. 70.00%), but the difference was not statistically significant (p = 0.358). There was a statistically significant difference in the incidence of complications between the two groups (P = 0.007); bleeding occurred more frequently in the Hot-Axios group (33.33% vs. 0%), whereas stent migration was more common in the Micro-Tech group (0% vs. 20.00%).

**Conclusions:**

In conclusion, endoscopic necrosectomy for infected WON using LAMS is feasible and effective. Hot-Axios may provide more secure fixation and better resistance to migration; however, it may carry a higher risk of bleeding.

## Introduction

The incidence of acute pancreatitis ranges from 3.4 to 73.4 cases per 100,000 persons per year worldwide and has been increasing at a rate of 2%−5% per year, with approximately 5,000–9,000 deaths reported each year [1–3]. According to the 2012 Atlanta International Guidelines, walled-off necrosis (WON) is a complication of severe acute pancreatitis characterized by the accumulation and encapsulation of necrotic material, which often manifests clinically as abdominal pain, gastrointestinal obstruction, and/or concurrent infections requiring intervention. Over the past decades, advances in the management of peripancreatic fluid collections (PFCs) have led to widespread adoption of the “step-up” approach [4–6]. In the first step, endoscopic or percutaneous drainage is performed. If drainage is insufficient to resolve the collection, the second step involves endoscopic or minimally invasive necrosectomy. If minimally invasive measures fail, surgical necrosectomy is considered as the third step. Compared with percutaneous or surgical strategies, endoscopic step-up therapy is less invasive and has become the primary treatment approach in most medical centers. WON contains varying proportions of solid necrotic components, and endoscopic drainage alone is often insufficient to achieve complete clearance of necrotic tissue. Studies have indicated that approximately 50% of WON cases ultimately require endoscopic debridement [7,8]. However, important clinical questions remain unresolved regarding endoscopic drainage and subsequent debridement for WON. In particular, uncertainty persists regarding (i) the optimal timing of endoscopic drainage, (ii) the choice of stent, (iii) when step-up necrosectomy should be initiated, (iv) when the lumen-apposing metal stent (LAMS) should be removed, and (v) whether additional double-pigtail plastic stents are needed after LAMS removal.

Lumen-apposing metal stents (LAMS) can be securely apposed between the gastrointestinal lumen and the necrotic cavity due to their double-flange design. Their wide lumen facilitates drainage and provides effective access for subsequent endoscopic debridement. To date, comparative evidence regarding different LAMS designs and their operational implications remains limited. Therefore, we retrospectively analyzed clinical data from WON patients who underwent drainage and necrosectomy with the assistance of two types of LAMS at our center, aiming to evaluate their effectiveness, safety, and detailed operational strategies.

## Methods

### Study design and participants

This single-center retrospective study included patients with infected WON who underwent endoscopic ultrasound (EUS)-guided LAMS placement and subsequent debridement at our hospital between August 2020 and June 2025. All endoscopic debridement procedures were performed by the same experienced senior attending physician (chief senior resident-level consultant). Written informed consent was obtained from all patients prior to treatment. The study protocol was approved by the Institutional Review Board of our center (NO. ZN-2024339–01) and initiated by the investigators. The date was first accessed for research purposes on 29/09/2024.

The inclusion criteria were as follows [9–11]: 1. Infected WON following acute pancreatitis. 2. Baseline imaging (CT or MRI) showed that the solid component accounted for >30% of the WON. 3. Patients underwent EUS-guided LAMS placement and debridement. 4. Necrotic lesions were tightly adherent to the gastric or duodenal wall (<1.5 cm). 5. Necrotic lesions were ≥3 cm in size, as measured on CT or MRI.

The exclusion criteria were as follows: 1. Pancreatic pseudocyst or noninfectious WON. 2. Underwent other types of endoscopic debridement procedures. 3. Abnormal coagulation function, pregnancy, or the presence of large blood vessels/varicose veins near the puncture site. 4. Refusal to undergo LAMS placement.

Antibiotics were routinely administered before and after the procedure. Follow-up assessments mainly included CT to measure lesion size at 10 months after endoscopic debridement, we completed follow-up of all patients on April 10, 2026. Any complications related to the management strategy were recorded when they occurred.

### Procedure technique

#### Hot-Axios LAMS (Boston Scientific Corp., Marlborough, Massachusetts, USA).

A liquid dark area was observed via ultrasonographic endoscopy, and flocculent deposits were observed in the cystic fluid near the gastric wall. Under ultrasonographic endoscopic guidance, the gastric wall was electrically incised with the anterior end of the Hot-Axios stent (diameter length: 10 15 mm), which allowed the release of the stent into the cystic cavity. The stent was well dilated, and a large amount of turbid fluid was observed flowing into the gastric cavity. At the end of the procedure, X-ray revealed that the stent was in place.

#### Micro-Tech LAMS (Micro-Tech Co., Ltd., Nanjing, China).

Ultrasonographic endoscopy revealed a fluid dark area with poor translucency of the cystic fluid near the gastric wall. After a COOK 19G puncture needle was used to puncture the cystic cavity, the needle core was removed, and thick liquid was observed during retraction. The guide wire was placed into the cystic cavity along with the puncture needle, and the sinus tract was incised with a gamma knife. Then, the puncture tract was dilated, the double mushroom-head metal stent (diameter × length: 16 × 20 mm) was placed along the guide wire, and a large amount of purulent liquid and solid necrotic material flowed into the gastric cavity. X-ray confirmed the optimal placement of the stent. The Micro-Tech LAMS (Micro-Tech Co., Ltd., Nanjing, China) is a novel LAMS described by Xu et al. [12]

#### Debridement through the LAMS.

A gastroscope was used to view the gastric cavity, the stent was confirmed to be in place, and a large amount of necrotic tissue was observed in the cystic cavity. Under loop visualization, foreign body forceps, retrieval baskets and snares were flexibly used to remove necrotic tissue from the gastric cavity until the necrotic cavity had been completely debrided, as confirmed endoscopically and by cross-sectional imaging (MRI or CT).

After the debridement of WON was completed, the metal stent was removed, and a guide wire was inserted into the sinus tract. The position of the guide wire was determined under X-ray fluoroscopy, and a double pigtail stent was placed over the guide wire to continue drainage.

#### Outcomes and definitions.

According to the revised Atlanta classification, WON is characterized by a collection of necrotic tissue in patients with a history of necrotizing pancreatitis. The primary outcomes were the necrosectomy success rate and the complication rate. Necrosectomy success was defined as complete resolution of WON without recurrence or rehospitalization within 6 months after the endoscopic debridement period. Complications—including bleeding, stent migration, and stent occlusion—were defined and graded according to the standard lexicon for endoscopic adverse events.

Secondary outcomes included: (1) time from onset of acute pancreatitis; (2) time from index drainage to first necrosectomy; (3) total number of necrosectomies required for WON resolution; (4) overall debridement time per patient; (5) duration of debridement of necrotic tissue; (6) LAMS retention time; and (7) drainage time after the last necrosectomy.

### Statistical analysis

Categorical variables are presented as counts (n) and percentages (%). Between-group differences were assessed using Fisher’s exact test. Continuous variables were assessed for normality using the Shapiro–Wilk test. Normally distributed variables are reported as mean(standard deviation, SD) and compared using Welch’s t-test. Non-normally distributed variables are reported as medians (interquartile range, IQR) and compared using the Mann–Whitney U test. Effect sizes were reported with 95% confidence intervals (CIs) where applicable. For binary categorical outcomes, effect sizes were expressed as odds ratios (OR) with 95% CIs. For normally distributed continuous outcomes, effect sizes were expressed as mean differences (95% CIs), and for non-normally distributed continuous outcomes, as median differences (95% CIs). All tests were two-sided, and statistical significance was set at P < 0.05. Statistical analyses were performed using SPSS (version 25; IBM, USA) and Python.

## Results

### Baseline characteristics

A total of 31 patients with WON were included: 21 in the Hot-Axios group and 10 in the Micro-Tech group. The mean age was 47.06 (16.86) years, 35.48% were female, and the median BMI was 22.00 (IQR 3.20). Etiologies of pancreatitis included gallstone pancreatitis (48.39%, 15/31), hyperlipidemic pancreatitis (29.03%,9/31), alcohol-related pancreatitis (5.45%, 2/31), and pancreatitis of unknown etiology (16.13%, 5/31). The mean size of the necrotic lesion was 12.77 (4.64) cm.

Clinical manifestations of infected WON included abdominal pain (60.00%), fever (19.35%), and both symptoms (12.90%). C-reactive protein levels were elevated (median 70.00 (IQR 157.70) mg/L). CT imaging demonstrated encapsulated necrosis, and gas shadows were present in some of the collections. Baseline characteristics were comparable between the Hot-Axios and Micro-Tech groups (Table 1 and Figs 1–10).

**Table 1:** Baseline characteristics of the Hot-Axios and Micro-Tech groups.

	Overall (n = 31)	Hot-Axios (n = 21)	Micro-Tech (n = 10)	P	Effect size*
Age, mean(SD), years	47.06(16.86)	47.71(17.20)	45.70(16.96)	0.762	2.01 (−11.44-15.47)
Sex, female, n(%)	11(35.48)	8(38.10)	3(30.00)	1.000	0.70(0.14-3.50)
BMI (kg/m^2^), median (IQR)	22.00(3.20)	22.20 (3.15)	21.40 (6.07)	0.811	0.80 (−3.80-2.60)
Etiology of acute pancreatitis, n (%)				0.279	–
Gallstones	15(48.39)	12(57.14)^a^	3(30.00)^a^		
Hyperlipidemia	9(29.03)	4(19.05)^a^	5(50.00)^a^		
Alcohol	2(5.45)	2(9.52)^a^	0^a^		
Others	5(16.13)	3(14.29)^a^	2(20.00)^a^		
Diameter, mean (SD), cm	12.77 (4.64)	12.68 (5.13)	12.95 (3.67)	0.883	−0.27 (−3.98-3.44)
C-reactive protein, median (IQR), mg/L	70.00(157.70)	107.00 (184.35)	65.00 (57.05)	0.421	42.00 (−47.70-134.00)
Clinical presentation, n (%)				0.185	–
Fever	6(19.35)	6(28.57)^a^	0^a^		
Pain	21(60.00)	13(61.90)^a^	8(80.00)^a^		
Fever and pain	4(12.90)	2(9.52)^a^	2(20.00)^a^		
Follow up, median (IQR), months	12.00 (1.50)	12.00 (3.00)	12.00 (1.75)	0.205	0.000 (0.000-2.500)

IQR, interquartile range; BMI: Body mass index; SD, standard deviation; CI, confidence interval. *: Effect sizes were reported with 95% CIs alongside corresponding P values: odds ratios (OR) for categorical outcomes, mean differences for normally distributed continuous variables, and median differences for non-normally distributed continuous variables. Superscripts (a, b) indicate statistical significance between groups as defined in the table.

**Fig 1:**
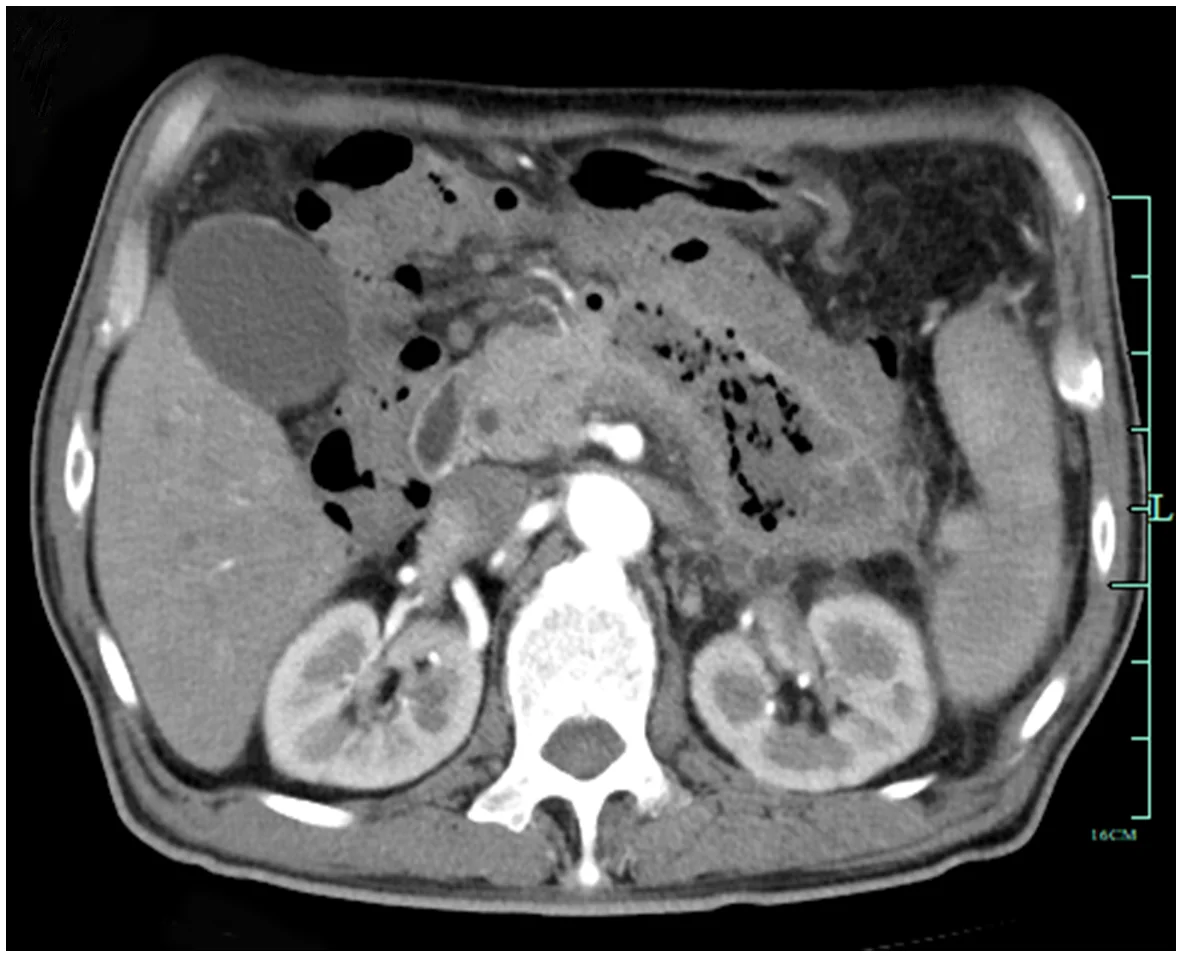
Computed tomography image of a walled-off necrosis.

**Fig 2:**
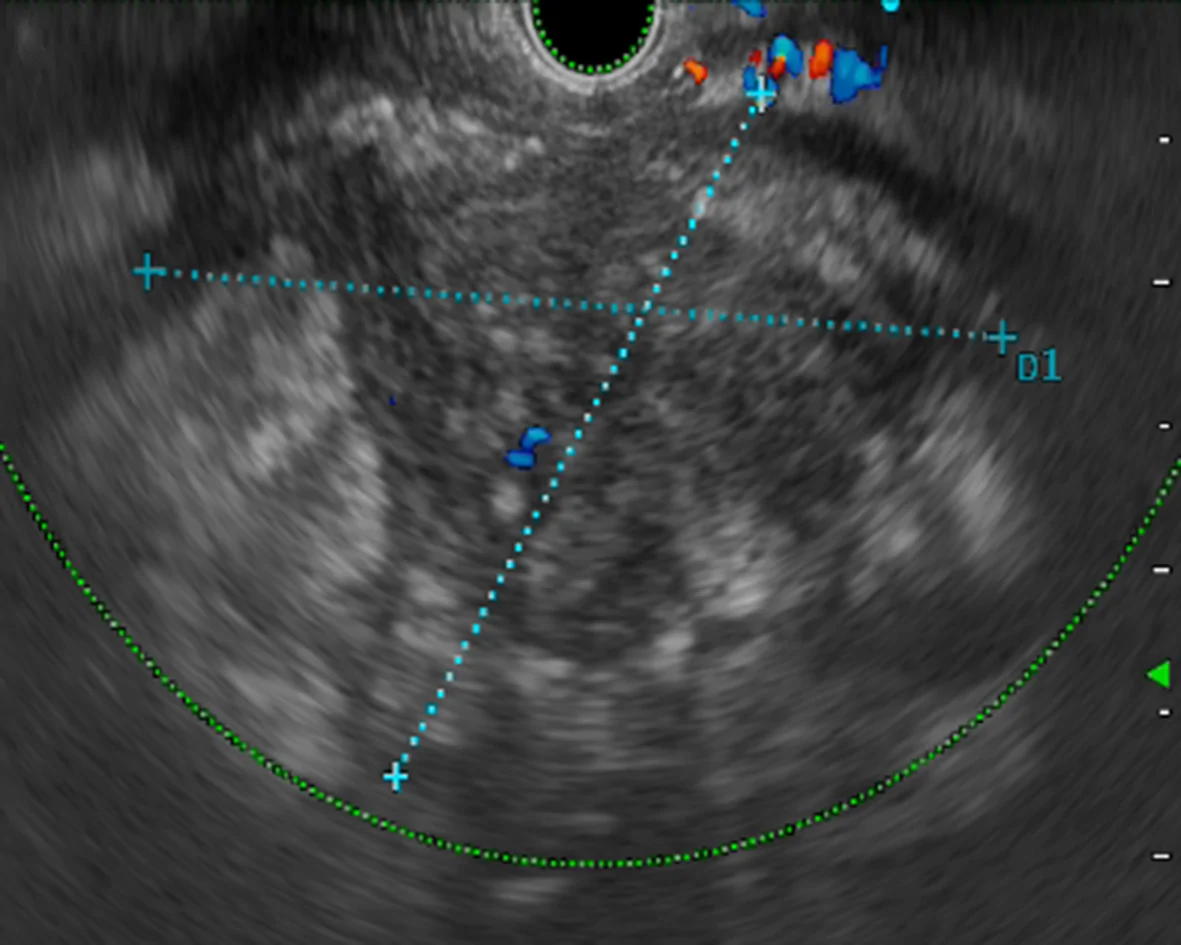
Endoscopic ultrasound image of a walled-off necrosis.

**Fig 3:**
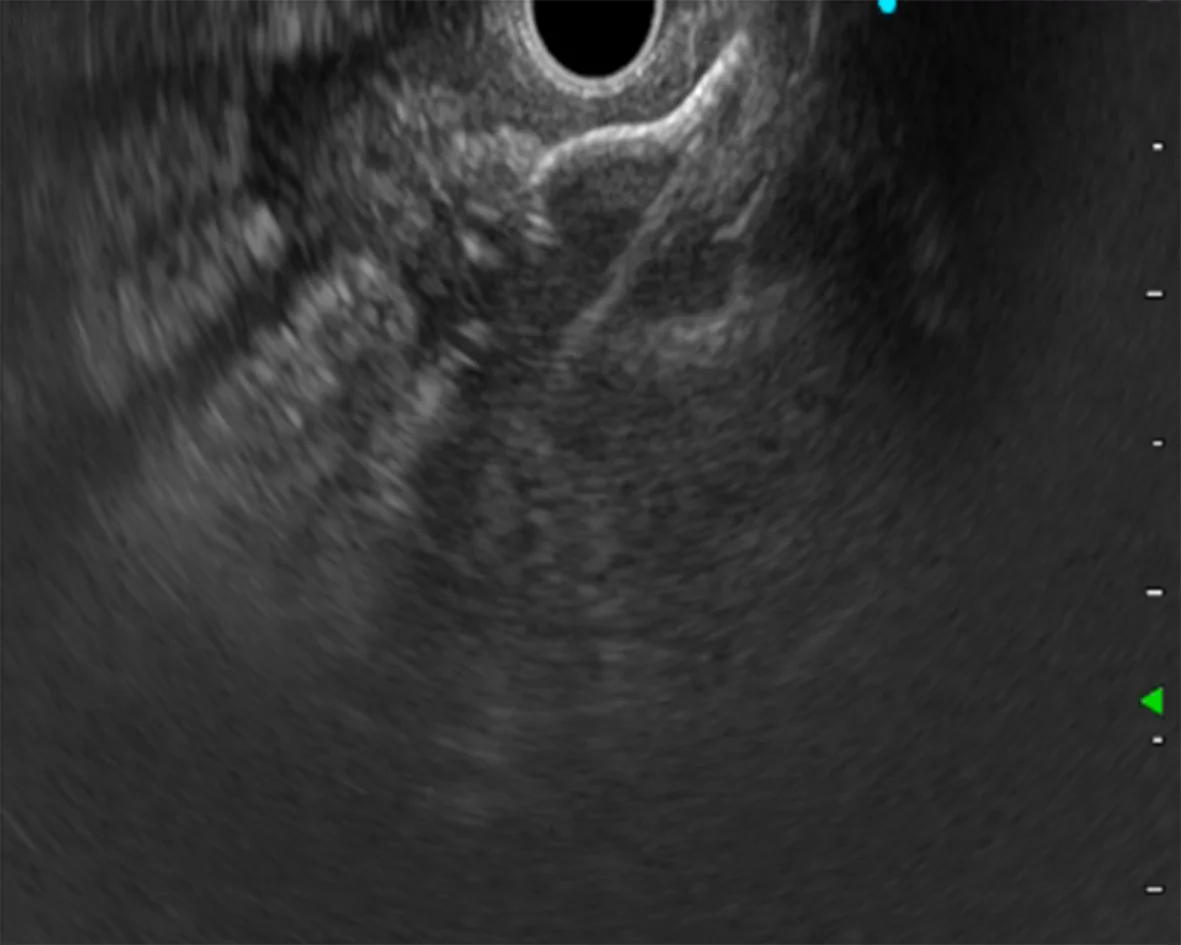
Endoscopic ultrasound-guided placement of a lumen-apposing metal stent.

**Fig 4:**
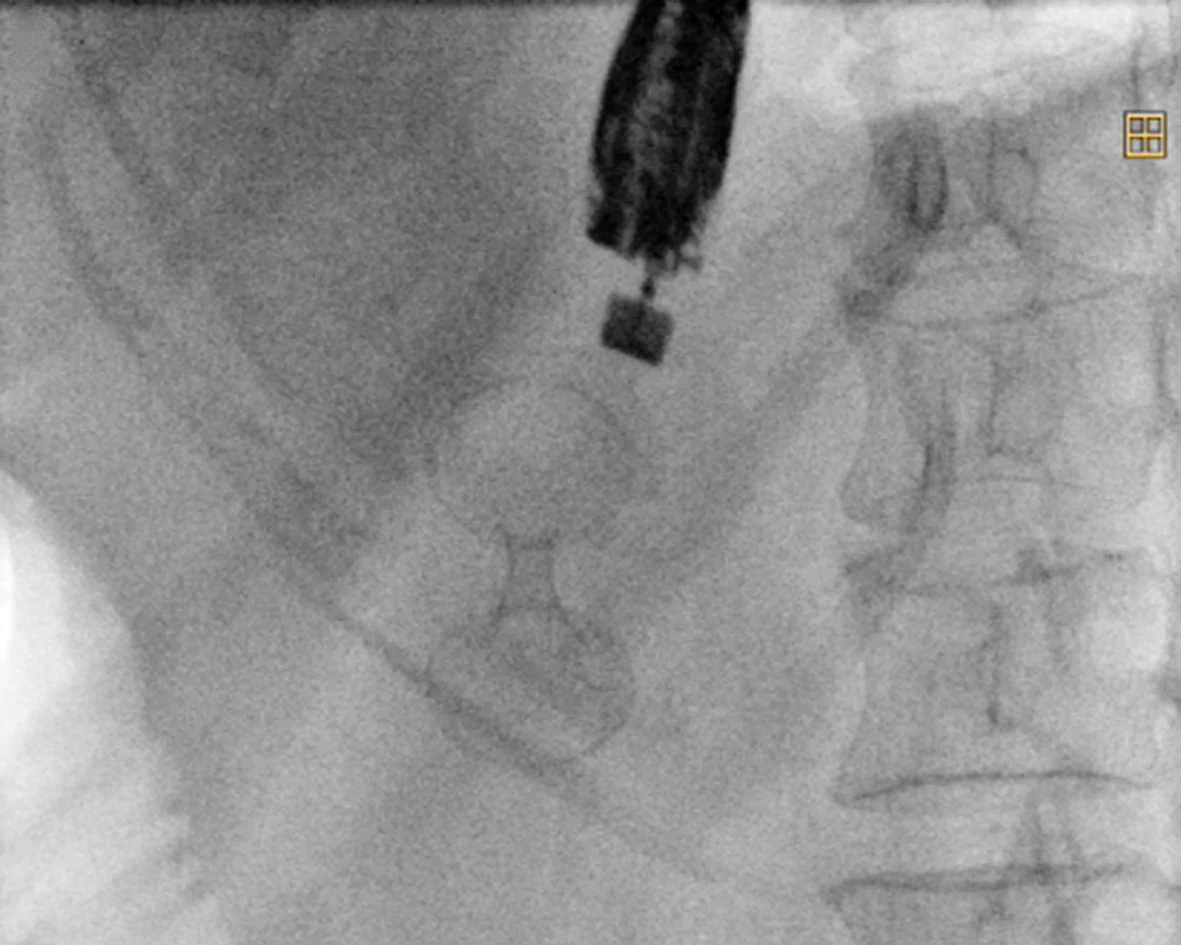
Fluoroscopy imaging of a lumen-apposing metal stent.

**Fig 5:**
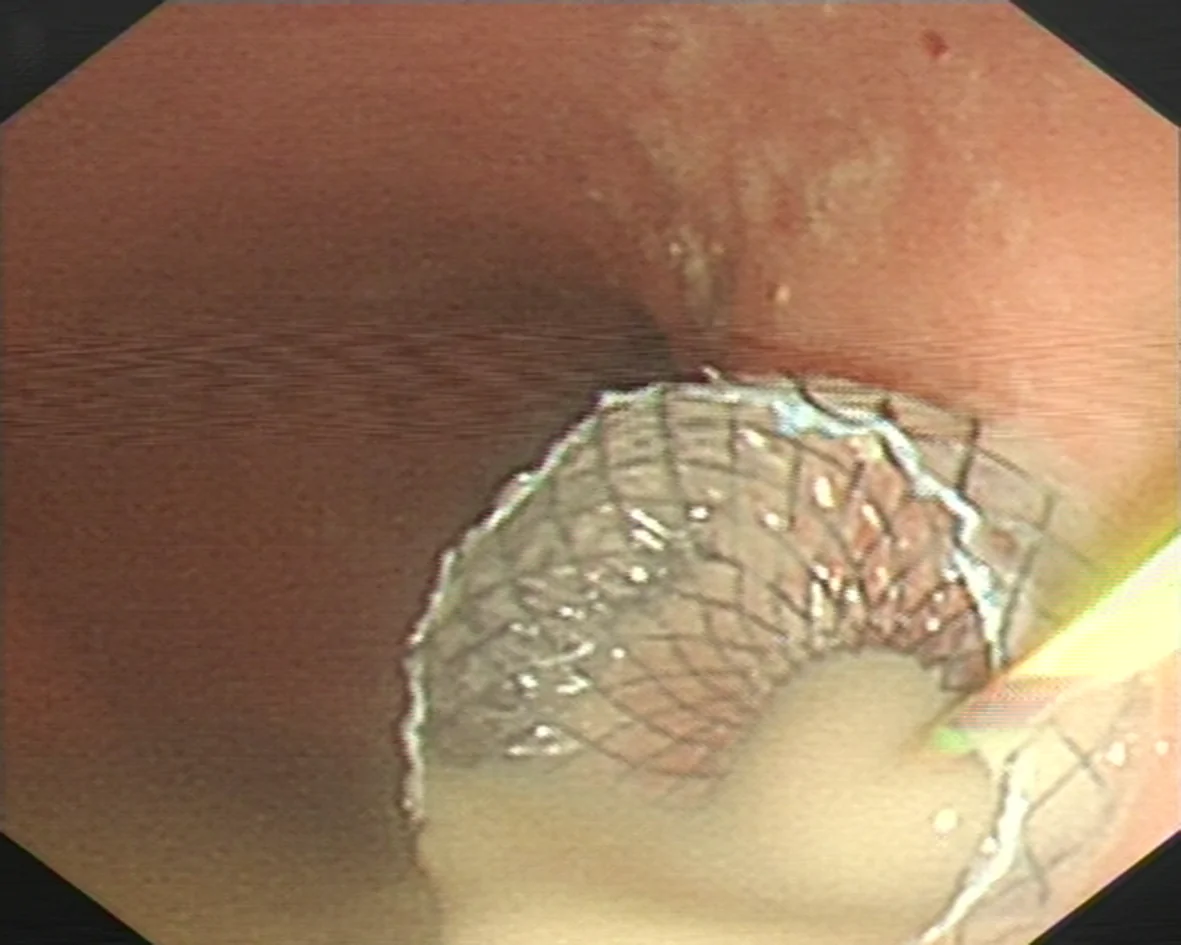
Endoscopic view showing purulent material draining through the lumen-apposing metal stent.

**Fig 6:**
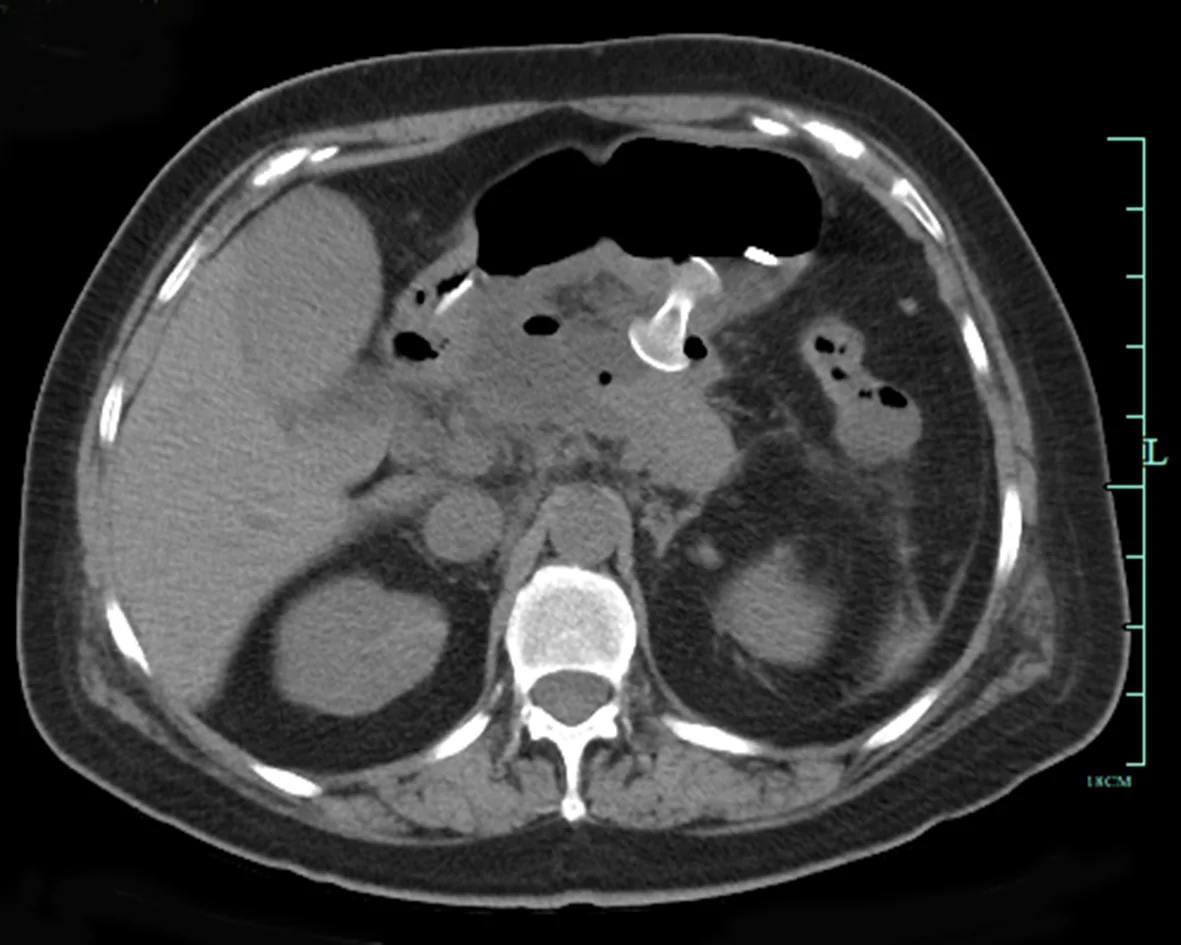
Computed tomography image demonstrating the lumen-apposing metal stent bridging the walled-off necrosis and the stomach.

**Fig 7:**
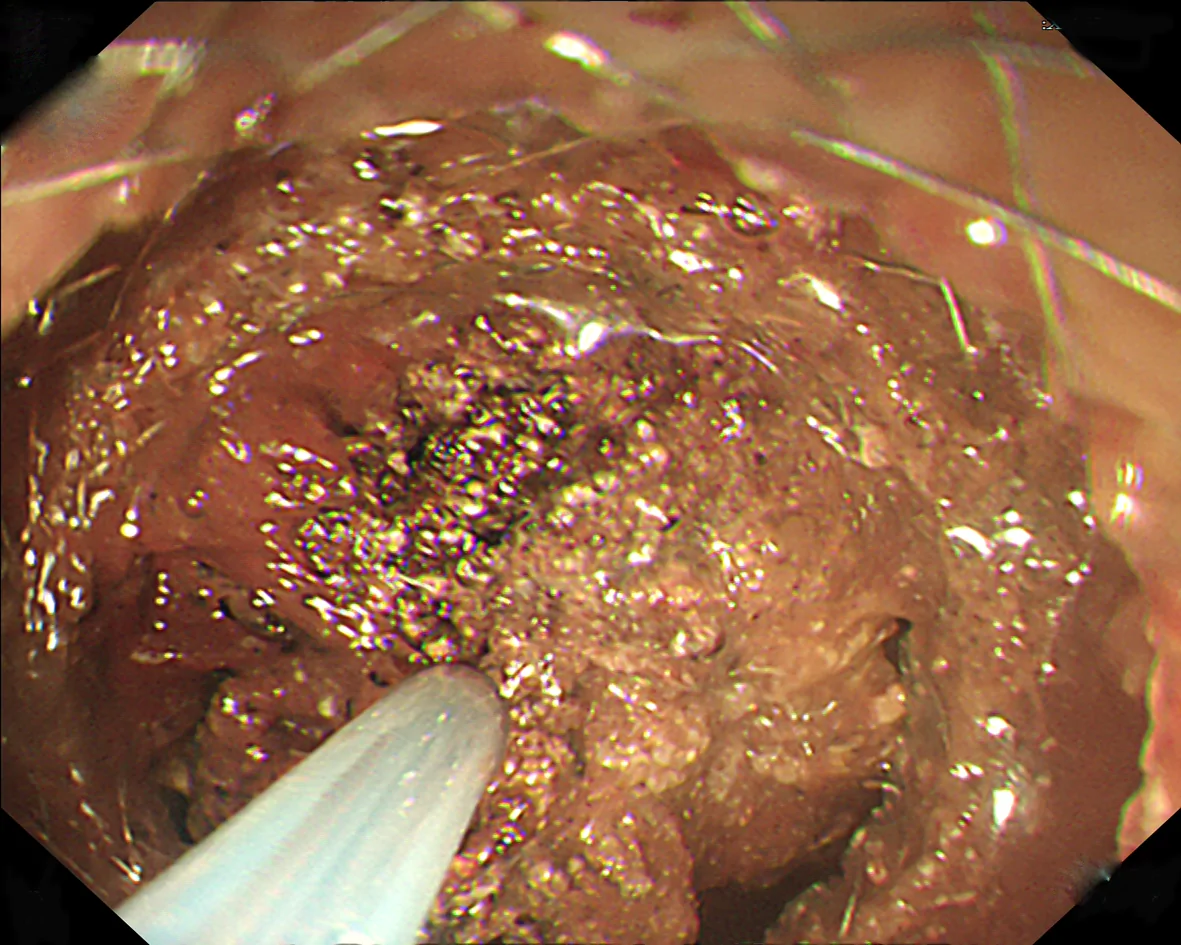
Endoscopic view of necrotic material removed through the lumen-apposing metal stent.

**Fig 8:**
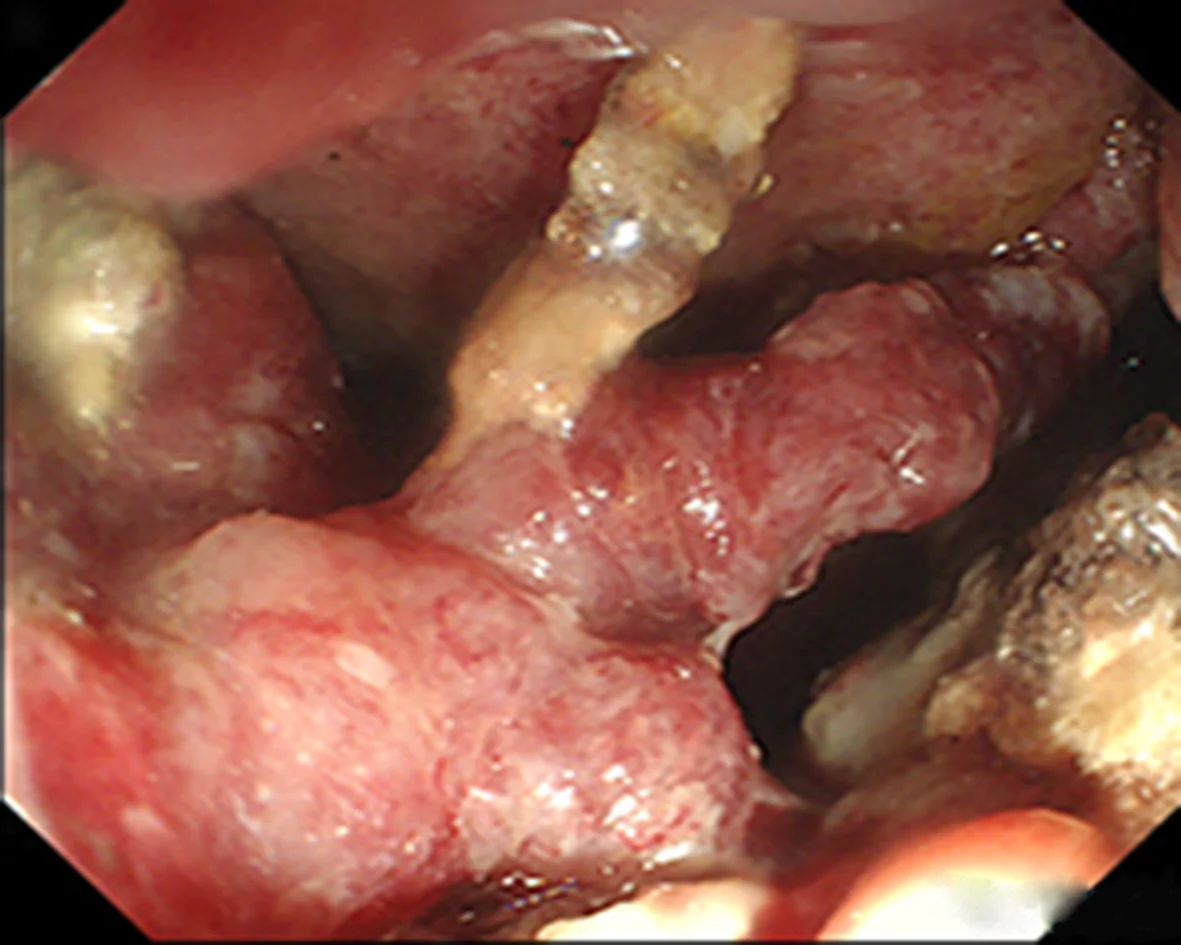
Endoscopic view showing granulation tissue within the cavity after debridement.

**Fig 9:**
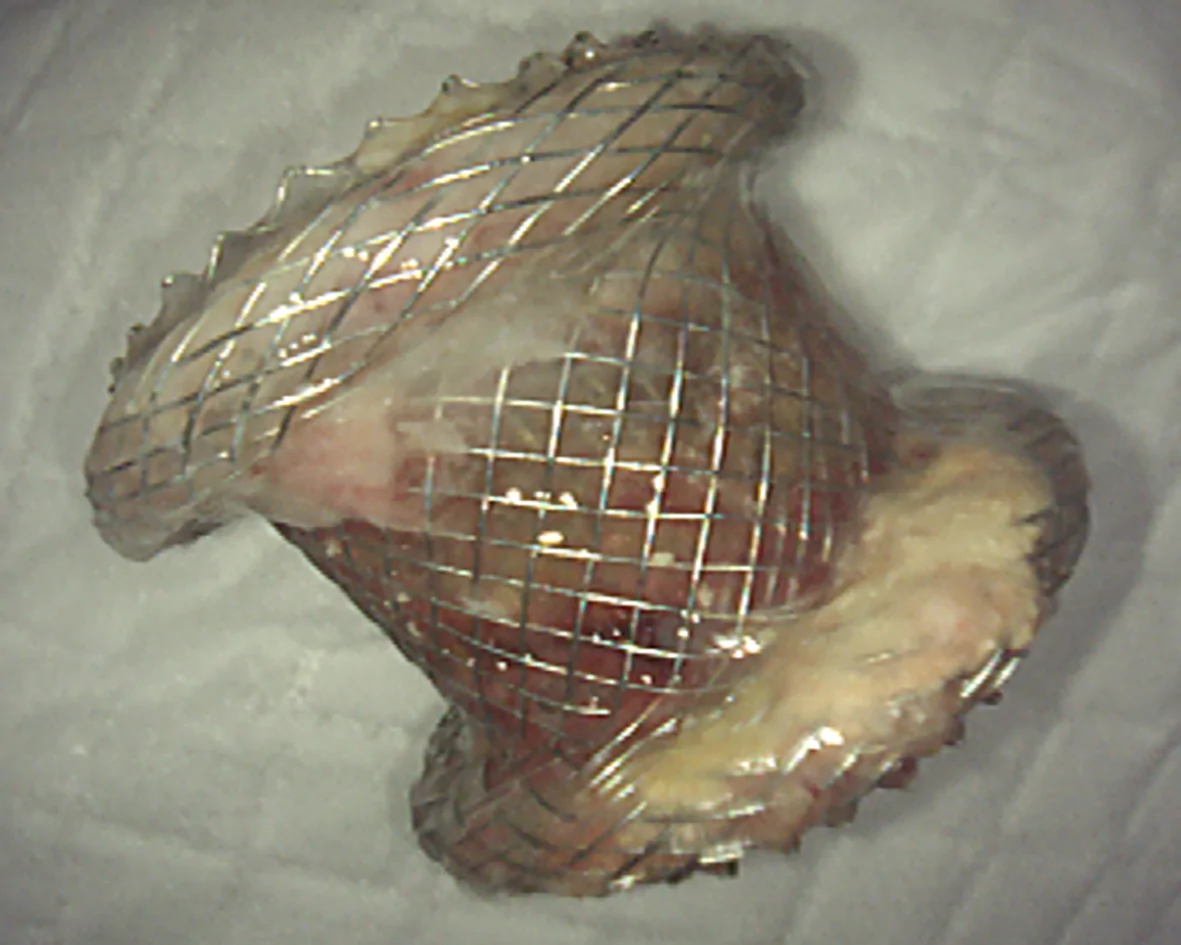
Photograph of the Hot-Axios lumen-apposing metal stent.

**Fig 10:**
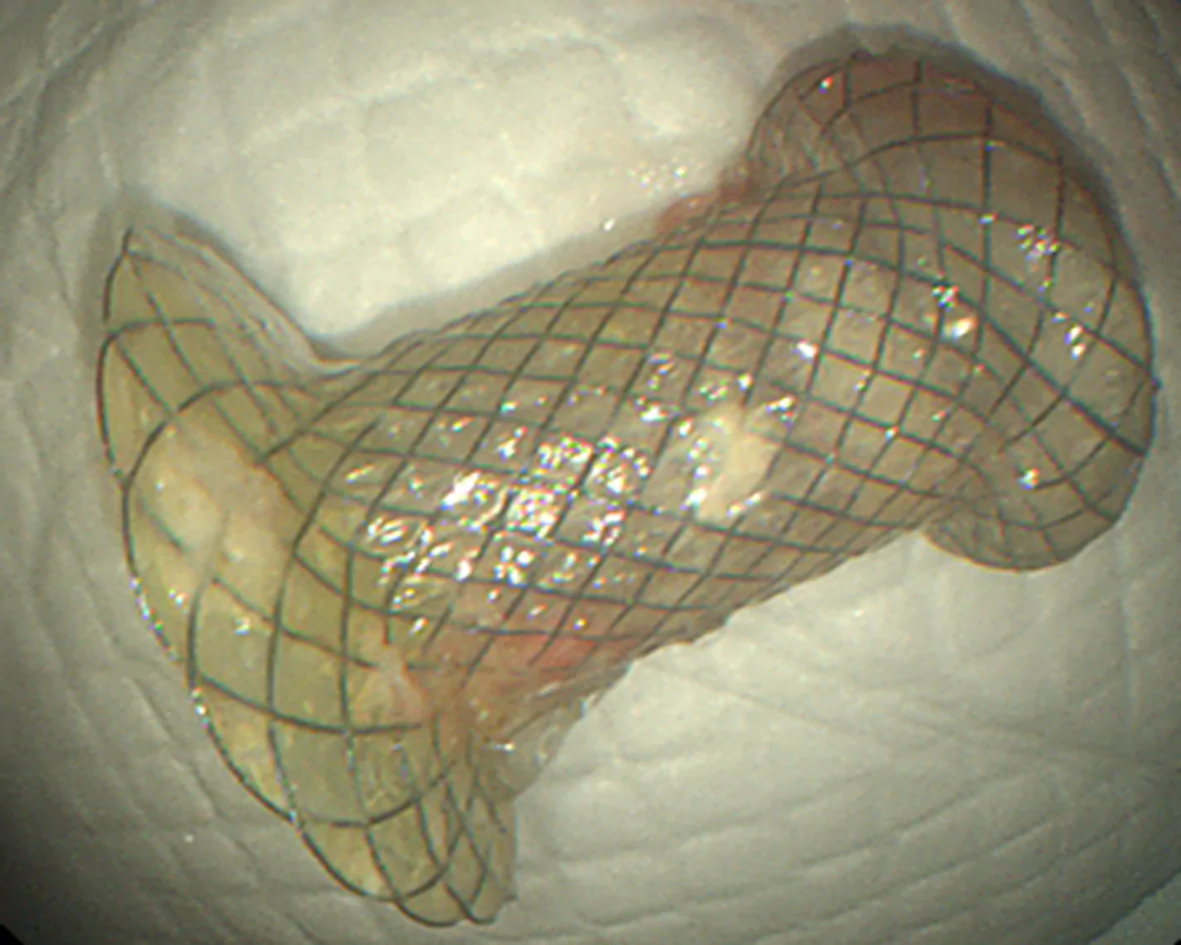
Photograph of the Micro-Tech lumen-apposing metal stent.

### Operating characteristics

All patients underwent LAMS placement after an interval of 1 month following acute pancreatitis onset, with a median of 1.00 (IQR 2.00) months. After LAMS remained in place for drainage (median 4.00 (IQR 3.00) days), abdominal CT was repeated to evaluate lesion size and the solid component prior to debridement.

Patients underwent debridement a median of 2.00 (IQR 2.00) times, with a total debridement time of 129.00 (IQR 176.00) minutes per case and a debridement duration of 11.00 (IQR 16.00) days. The average LAMS retention time was 21.32 (SD 11.80) days. After LAMS removal, a double-pigtail stent was used to continue drainage for 41.71 (IQR 44.21) days. Operating characteristics did not differ significantly between the two groups (Table 2).

**Table 2:** Procedural details.

	Overall(n = 31)	Hot-Axios (n = 21)	Micro-Tech (n = 10)	P	Effect size*
Necrosectomy success, n (%)	25 (80.65%)	18(85.71%)	7(70.00%)	0.358	2.57 (0.42-15.92)
Time from onset of acute pancreatitis, months, median (IQR)	1.00(2.00)	1.00 (1.50)	1.50 (2.00)	0.736	−0.50 (−1.51-1.00)
Time from index drainage to first necrosectomy, days, median (IQR)	4.00(3.00)	4.00 (4.00)	5.00 (2.75)	0.151	−1.00 (−3.50-1.00)
Number of necrosectomies, median (IQR)	2.00(2.00)	2.00 (2.00)	2.50 (1.25)	0.951	−0.50 (−1.00-1.00)
Overall debridement time per patient, minutes, median (IQR)	129.00(176.00)	129.00 (216.00)	122.00 (89.25)	0.917	7.00 (−93.00-121.00)
Duration of debridement of necrotic tissue, days, median (IQR)	11.00(16.00)	10.00 (14.00)	14.50 (20.75)	0.299	−4.50 (−17.00-7.00)
Days to stent removal, mean (SD), days	21.32 (11.80)	20.62 (12.38)	22.80 (10.94)	0.638	−2.18 (−11.58-7.21)
Time of drainage after last necrosectomy, days, median (IQR)	41.71(44.21)	36.00 (57.00)	6.00 (57.75)	0.241	30.00(−36.53-45.50)
Adverse event rate, n (%)				0.007	–
Bleeding	7 (22.58)	7(33.33)^a^	0^b^		
Stent migration	2 (6.45)	0^a^	2(20.00)^b^		
Stent occlusion	1 (3.23)	0^a^	1(10.00)^a^		
No adverse events	21 (67.74)	14(66.67)^a^	7(70.00)^a^		

IQR, interquartile range; SD, standard deviation; CI, confidence interval. *: Effect sizes were reported with 95% CIs alongside corresponding P values: odds ratios (OR) for categorical outcomes, mean differences for normally distributed continuous variables, and median differences for non-normally distributed continuous variables. Superscripts (a, b) indicate statistical significance between groups as defined in the table.

### Outcomes

#### Overall success and management of failures.

The overall rate of successful necrosectomy assisted by LAMS in our center was 80.65% (25/31). The success rate was numerically higher in the Hot-Axios group than in the Micro-Tech group (85.71% vs. 70.00%), but the difference was not statistically significant (p = 0.358), effect sizes with 95% CIs are provided in Table 2.

Among the six failed cases, three involved Hot-Axios stents removed due to bleeding complications. One case involved Micro-Tech stent migration, and one involved Micro-Tech stent occlusion. These five cases proceeded to endoscopic plastic stent drainage and additional debridement. One patient with displaced Micro-Tech stent underwent surgical debridement after stent removal. Ultimately, all patients achieved resolution of WON with completion of necrosectomy treatment (Table 2 and Fig 1).

#### Complications.

Bleeding during debridement occurred in 7 cases (33.33%, 7/21) in the Hot-Axios group and was successfully managed using endoscopic hemostasis, including titanium clip placement (3 cases) or topical norepinephrine application (4 cases). Bleeding occurred at days 9, 10, 12, 14, 18, 20, and 21 after stent implantation. Four cases continued debridement through the original Hot-Axios stent after hemostasis, whereas three cases underwent stent removal followed by debridement via endoscopic plastic stent placement.

Micro-Tech stent migration occurred in 2 cases (6.45%, 2/31). Both were successfully managed with endoscopic stent removal; one required conversion to surgical debridement, while the other completed debridement later using plastic stent placement. Micro-Tech stent occlusion occurred in 1 case (3.23%, 1/31); the stent was removed endoscopically, and debridement was completed via plastic stent placement.

Overall, bleeding incidence was significantly higher in the Hot-Axios group (33.33% vs. 0, P = 0.007), whereas stent migration incidence was significantly higher in the Micro-Tech group (0 vs. 20.00%, P = 0.007) (Table 2 and Fig 1).

## Discussion

Infected WON commonly presents with abdominal pain, fever, or gastrointestinal obstruction and typically requires timely clinical intervention. Several studies suggest that mortality and complication rates are lower when drainage/puncture is delayed until the capsule wall matures (approximately 4 weeks) [4,13]. Meta-analyses have further supported the benefit of delayed intervention [14,15]. Nonetheless, early intervention is sometimes unavoidable in critically ill patients due to rapid disease progression and severe infection.

Equally important is the uncertainty regarding the optimal timing of escalation to step-up endoscopic necrosectomy. Chandrasekhara et al. [9] reported that approximately half of WON patients require additional debridement. Lesion characteristics associated with further debridement include lesions >10 cm, necrotic material extending to the paracolon, and collections containing >30% solid components. In such cases, early debridement may lead to early clinical resolution. Conversely, Wu et al. [16] reported that the clinical success rate after LAMS debridement was 75% at 6 months and that there were no significant differences in clinical success, debridement time, complications, length of hospital stay, or overall prognosis between patients treated within 1 week versus those treated after 1 week following stent placement. They also observed a lower rate of additional debridement interventions in the later group (26.8% vs. 8.3%, P = 0.036). Another retrospective study suggested that delayed debridement reduced hospitalization duration and the total number of debridement sessions [17]. Given that 86.4% of experts do not recommend simultaneous drainage and debridement, debridement is in fact recommended for 3–21 days after drainage [18].

Plastic stents are frequently used as the first-line option for endoscopic drainage of PFCs such as pseudocysts; however, the clinical success rate of WON drainage with plastic stents is only about 47%–50% [7,8]. At present, the comparative safety and effectiveness of plastic stents versus LAMS for PFC management remain incompletely defined. A retrospective study [19] including 106 patients who underwent DPS (Duct plastic stent) and 86 patients who underwent LAMS showed a lower clinical success rate with the DPS strategy (p = 0.001), and plastic stenting remained the sole negative predictor for successful WON resolution. In contrast, Kaveh et al. [20] reported no significant differences between DPS and LAMS groups regarding adverse event rates (4% vs. 6%, P = 0.24), clinical success (60% vs. 61%, P = 0.94), or need for reintervention (11% vs. 13%, P = 0.72) in their retrospective cohort of 89 patients.

Notably, in many retrospective studies, LAMS tends to be preferred for collections with a higher burden of solid necrotic components due to superior drainage capability and suitability for subsequent debridement. Conversely, collections with fewer solid components may be managed using plastic stents, which are less expensive but may be more susceptible to small puncture-related trauma. This selection bias can reduce the apparent performance of LAMS in some comparisons. Therefore, it is critical to clearly quantify the proportion of solid necrosis when evaluating differences between LAMS and plastic stents, as reducing bias improves the robustness and generalizability of conclusions.

Relevant meta-analyses [21] reported no significant differences in treatment success, overall complication rate, bleeding rate, new organ failure rate, or mortality rate, although LAMS treatment duration was shorter than that with plastic stents for WON. In another study from the same group, plastic stent drainage for necrotic lesions containing >33% solid necrotic material was associated with post-puncture systemic inflammatory response syndrome (SIRS) and new-onset organ failure. Accordingly, LAMS drainage was recommended for necrotic lesions with >33% solid necrotic material [10]. Moon et al. [22] found that the number of debridement sessions required to achieve clinical success did not differ significantly between LAMS and plastic stents; however, clinical success was higher in the LAMS group at 8 weeks (100% vs. 73.9%, P = 0.03), even though both groups achieved debridement completion in all patients. Compared with DPS, LAMS achieved earlier success and shorter treatment time, while bleeding and other complications were not significantly different (52.2% vs. 26.1%, P = 0.13). Weigand et al. [23] reported that in 9 WON patients treated with LAMS, clinical success was 100%, with LAMS drained for 2–12 weeks after debridement and no complications. Taken together, these findings highlight the need for multicenter prospective randomized controlled trials to further validate evidence.

A recent study [12] reported that minimally invasive placement of Micro-Tech stents for WON achieved clinical success rates ranging from 76.6% to 96.5%, comparable to Hot-Axios stenting (89%–96%). In our study, the clinical success rate of WON debridement was higher in the Hot-Axios group than in the Micro-Tech group, although the difference was not statistically significant (85.71% vs. 70.00%, P = 0.358). Bleeding is among the most common complications, with reported incidence ranges of 15%–25%, typically occurring approximately 9–26 days after stent placement; most bleeding can be managed conservatively [24–26]. In our cohort, all 7 bleeding cases were treated conservatively. In 4 patients, bleeding was controlled and debridement continued using the original Hot-Axios stent after hemostasis. Potential mechanisms include inflammation-related erosion of blood vessels, traction on granulation tissue and vessels during debridement, and prolonged friction between the lesion and the stent [24,25]. In practice, these mechanisms are often difficult to distinguish and bleeding is frequently multifactorial. Some literature suggests that Hot-Axios stent design—specifically the rigid terminal spikes—may scrape the contralateral cavity wall during stent contraction, leading to vessel erosion and subsequent bleeding [24,27]. Therefore, prevention should target multiple aspects: careful debridement to avoid vessels and excessive traction, and systematic follow-up after stent placement. Regular CT assessment can help evaluate necrotic debris around the stent and the status of adjacent vessels, while timely stent removal after adequate drainage may reduce risk of encapsulation by granulation tissue. Concurrently, continued improvements in stent technology are warranted.

There remains controversy regarding the optimal timing of LAMS removal. Delayed removal beyond 4 weeks may increase the risk of delayed bleeding, stent embedding, and other complications [13,28]. However, Stigliano et al. [29] reported no association between LAMS indwelling >4 weeks and complications (88.9% vs. 69.9%, P = 0.44), though higher complication rates were associated with larger LAMS size (>15 mm) and head location. A recent retrospective study [30] found that removal after 4 weeks did not increase complication rates but reduced clinical success (70.0% vs. 96.4%, P = 0.03), and no predictors of complications were identified. Amato et al. [31] conducted a subgroup analysis based on the LAMS insertion-to-removal interval (early <4 weeks vs. late >4 weeks). The results showed no statistically significant differences between the early and late groups in terms of adverse events (AEs) (5% vs. 10%, respectively; p = 0.19) or recurrence rates (8% vs. 3%, respectively; p = 0.17). Therefore, the impact of the timing of LAMS removal may be smaller than previously thought. In our center, LAMS was left in place for 21.32 (11.80) days on average and removed within 4 weeks to minimize bleeding, embedding, and related risks. In cases of refractory or very large WON, stent dwell time may be prolonged appropriately, with close monitoring for complications. Before LAMS removal, we routinely perform cross-sectional imaging to assess the resolution of peripancreatic fluid collections and necrotic debris, as recommended by the guidelines [11]. Because most current evidence comes from single-center retrospective studies, further prospective multicenter studies are needed to enhance generalizability.

Whether double pigtail plastic stents (DPS) should be kept for extended periods after LAMS removal remains unclear. It has been reported that patients with WON frequently have pancreatic duct injury, including disconnected pancreatic duct syndrome (DPDS), duct disruption, persistent pancreatic leakage, and PFC recurrence, which occurs in approximately 16%–23% of WON patients [32]. Replacement of DPS with long-term drainage after LAMS placement has been associated with reduced PFC recurrence (0%–5% vs. 15.4%–37%) [33–35]. In our study, DPS was removed after continuous drainage for 41.71 days. At 6-month follow-up, no further debridement intervention was required. Overall, DPS replacement after LAMS extraction may be recommended for WON, particularly in patients with DPDS. However, the success rate of DPS placement after LAMS removal has been reported to be low, ranging from 44% to 74% [34,35]. potentially because the WON lumen collapses immediately after LAMS removal, narrowing the operative space for PDS insertion. Mukai et al. [33] introduced a “puzzle ring replacement technique” that improved success from 61.8% to 94.4%. In our study, the technical success rate of DPS placement along the guidewire reached 100% with assistance from fluoroscopic guidance.

This study has several strengths. First, it provides an early comparative assessment of the efficacy and safety of Hot-Axios and Micro-Tech LAMS for infected WON. Second, all procedures were performed by the same experienced senior endoscopist, which helped reduce operator-related variability. Several limitations should be considered. The study was retrospective and conducted at a single center, which may limit generalizability. The sample size was small and the group sizes were unequal. In addition, despite using appropriate statistical tests and reporting effect sizes with 95% CIs, unmeasured confounding may still exist. Therefore, our findings should be interpreted cautiously, and prospective multicenter studies are warranted to validate these results.

## Conclusion

LAMS is a safe and effective tool for endoscopic debridement of infected pancreatic necrosis. Compared with the Micro-Tech stent, the Hot-Axios stent may provide more secure fixation and greater resistance to displacement; however, it could be associated with an increased risk of bleeding. Given the limitations of this study, the accuracy of these findings should be confirmed in future prospective randomized controlled trials.

## References

[1] Xiao AY, Tan MLY, Wu LM, Asrani VM, Windsor JA, Yadav D, et al. Global incidence and mortality of pancreatic diseases: a systematic review, meta-analysis, and meta-regression of population-based cohort studies. Lancet Gastroenterol Hepatol. 2016;1(1):45–55. doi: 10.1016/S2468-1253(16)30004-8 28404111

[2] Peery AF, Crockett SD, Murphy CC, Lund JL, Dellon ES, Williams JL. Burden and cost of gastrointestinal, liver, and pancreatic diseases in the United States: update 2018. Gastroenterology. 2019;156(1):254-272.e11. 10.1053/j.gastro.2018.08.063 PMC6689327 30315778

[3] Tenner S, Vege SS, Sheth SG, Sauer B, Yang A, Conwell DL, et al. American College of gastroenterology guidelines: management of acute pancreatitis. Am J Gastroenterol. 2024;119(3):419–37. doi: 10.14309/ajg.0000000000002645 38857482 PMC13221274

[4] Baron TH, DiMaio CJ, Wang AY, Morgan KA. American Gastroenterological Association clinical practice update: management of pancreatic necrosis. Gastroenterology. 2020;158(1):67-75.e1. 10.1053/j.gastro.2019.07.064 31479658

[5] Valentin C, Le Cosquer G, Tuyeras G, Culetto A, Barange K, Hervieu P-E, et al. Step-up approach for the treatment of infected necrotising pancreatitis: real life data from a single-centre experience with long-term follow-up. BMC Gastroenterol. 2024;24(1):213. doi: 10.1186/s12876-024-03289-6 38943052 PMC11214239

[6] Tran Z, Xu J, Verma A, Ebrahimian S, Cho NY, Benharash P, et al. National trends and clinical outcomes of interventional approaches following admission for infected necrotizing pancreatitis in the United States. J Trauma Acute Care Surg. 2023;94(5):665–71. doi: 10.1097/TA.0000000000003934 36805574

[7] Karstensen JG, Novovic S, Hansen EF, Jensen AB, Jorgensen HL, Lauritsen ML, et al. EUS-guided drainage of large walled-off pancreatic necroses using plastic versus lumen-apposing metal stents: a single-centre randomised controlled trial. Gut. 2023;72(6):1167–73. doi: 10.1136/gutjnl-2022-328225 36446550

[8] Boxhoorn L, Verdonk RC, Besselink MG, Boermeester M, Bollen TL, Bouwense SA, et al. Comparison of lumen-apposing metal stents versus double-pigtail plastic stents for infected necrotising pancreatitis. Gut. 2023;72(1):66–72. doi: 10.1136/gutjnl-2021-325632 35701094

[9] Chantarojanasiri T, Ratanachu-Ek T, Isayama H. When should we perform endoscopic drainage and necrosectomy for walled-off necrosis?. J Clin Med. 2020;9(12). 10.3390/jcm9124072 PMC7767133 33348675

[10] Bang JY, Wilcox CM, Navaneethan U, Hawes R, Varadarajulu S. Impact of endoprosthesis type on inflammatory response in patients undergoing endoscopic drainage of pancreatic fluid collections. Dig Endosc. 2024;36(2):195–202. doi: 10.1111/den.14565 37039707

[11] Stefanovic S, Adler DG, Arlt A. International consensus recommendations for safe use of LAMS for on- and off-label indications using a modified Delphi process. Journal Title Abbreviation. 2024;119(4):671–81. 10.14309/ajg.0000000000002571 37934190

[12] Xu N, Li L, Su S, Zhao D, Xiang J, Wang P, et al. A novel lumen-apposing metal stent for endoscopic drainage of symptomatic pancreatic fluid collections: a retrospective study. Endosc Ultrasound. 2024;13(1):40–5. doi: 10.1097/eus.0000000000000039 38947119 PMC11213609

[13] Arvanitakis M, Dumonceau J-M, Albert J, Badaoui A, Bali MA, Barthet M, et al. Endoscopic management of acute necrotizing pancreatitis: European Society of Gastrointestinal Endoscopy (ESGE) evidence-based multidisciplinary guidelines. Endoscopy. 2018;50(5):524–46. doi: 10.1055/a-0588-5365 29631305

[14] Nakai Y, Shiomi H, Hamada T, Ota S, Takenaka M, Iwashita T, et al. Early versus delayed interventions for necrotizing pancreatitis: a systematic review and meta-analysis. DEN Open. 2022;3(1):e171. doi: 10.1002/deo2.171 36247314 PMC9549879

[15] Yang Y, Zhang Y, Wen S, Cui Y. The optimal timing and intervention to reduce mortality for necrotizing pancreatitis: a systematic review and network meta-analysis. World J Emerg Surg. 2023;18(1):9. doi: 10.1186/s13017-023-00479-7 36707836 PMC9883927

[16] Wu S, Dou X, Li N, Zhu H, Wang L, Liu M, et al. Postponed endoscopic necrosectomy results in a lower rate of additional intervention for infected walled-off necrosis. Sci Rep. 2024;14(1):11610. doi: 10.1038/s41598-024-61675-2 38773218 PMC11109209

[17] Pawa R, Dorrell R, Clark C, Russell G, Gilliam J, Pawa S. Delayed endoscopic necrosectomy improves hospital length of stay and reduces endoscopic interventions in patients with symptomatic walled-off necrosis. DEN open. 2023;3(1):e162. 10.1002/deo2.162 PMC9453323 36090191

[18] Guo J, Saftoiu A, Vilmann P, Fusaroli P, Giovannini M, Mishra G, et al. A multi-institutional consensus on how to perform endoscopic ultrasound-guided peri-pancreatic fluid collection drainage and endoscopic necrosectomy. Endosc Ultrasound. 2017;6(5):285–91. doi: 10.4103/eus.eus_85_17 29063871 PMC5664848

[19] Siddiqui AA, Kowalski TE, Loren DE, Khalid A, Soomro A, Mazhar SM, et al. Fully covered self-expanding metal stents versus lumen-apposing fully covered self-expanding metal stent versus plastic stents for endoscopic drainage of pancreatic walled-off necrosis: clinical outcomes and success. Gastrointest Endosc. 2017;85(4):758–65. doi: 10.1016/j.gie.2016.08.014 27566053

[20] Khodakaram K, Bratlie SO, Hedenström P, Sadik R. Equivalent efficacy and safety of plastic stents and lumen-apposing metal stents in the treatment of peripancreatic fluid collections: a prospective cohort study. Ann Gastroenterol. 2024;37(3):362–70. doi: 10.20524/aog.2024.0873 38779636 PMC11107401

[21] Bang JY, Wilcox CM, Navaneethan U, Hawes RH, Varadarajulu S. Treatment of walled-off necrosis using lumen-apposing metal stents versus plastic stents: a systematic review and meta-analysis of data from randomized trials. Endoscopy. 2024;56(3):184–95. doi: 10.1055/a-2169-0362 37673106

[22] Moon JH, Park SW, Lee YN, Lee SH, Kim S-H, Lee DW, et al. A comparison of novel electrocautery-enhanced lumen-apposing metal stents and plastic stents in endoscopic ultrasound-guided drainage of infected walled-off necrosis: a multicenter randomized study. Endoscopy. 2024;56(12):926–37. doi: 10.1055/a-2342-1140 38857618 PMC11716544

[23] Weigand K, Mehrl A, Goessmann H, Mueller M, Kandulski A. Endoscopic necrosectomy of walled-off necrosis following severe pancreatitis using a hot AxiosTM stent - a case series. Digestive diseases (Basel, Switzerland). 2019;:1–4. 10.1159/000503991 31726460

[24] Mangiavillano B, Lakhtakia S, Samanta J, Auriemma F, Vargas-Madrigal J, Arcidiacono PG, et al. Lumen-apposing metal stents for the treatment of pancreatic and peripancreatic fluid collections and bleeding risk: a propensity matched study. Endoscopy. 2024;56(4):249–57. doi: 10.1055/a-2219-3179 38237633

[25] Dell’Anna G, Lavalle S. Clinical, radiological, and endoscopic features of pancreatic pseudocyst and walled-off necrosis: how to diagnose and how to drain them. 2025;14(21). 10.3390/jcm14217818 PMC12608859 41227213

[26] Maatman TK, Heimberger MA, Lewellen KA, Roch AM, Colgate CL, House MG, et al. Visceral artery pseudoaneurysm in necrotizing pancreatitis: incidence and outcomes. Can J Surg. 2020;63(3):E272–7. doi: 10.1503/cjs.009519 32436687 PMC7829015

[27] Rimbaş M, Pizzicanella M, Larghi A. EUS-guided treatment of WON using lumen-apposing metal stents: protocol standardisation based on the occurrence of natural healing processes. Gut. 2019;68(7):1334–5. doi: 10.1136/gutjnl-2018-316994 30030302

[28] Bang JY, Hawes RH, Varadarajulu S. Lumen-apposing metal stent placement for drainage of pancreatic fluid collections: predictors of adverse events. Gut. 2020;69(8):1379–81. doi: 10.1136/gutjnl-2019-320539 32066622 PMC7398475

[29] Stigliano S, Marocchi G, Baldaro F, Neri B, Del Vecchio Blanco G, Troncone E, et al. Timing of lumen-apposing metal stents removal in pancreatic fluid collections: Could we go beyond?. Pancreatology. 2024;24(8):1252–6. doi: 10.1016/j.pan.2024.10.011 39488448

[30] Willems P, Esmail E, Paquin S, Sahai A. Safety and efficacy of early versus late removal of LAMS for pancreatic fluid collections. Endosc Int Open. 2024;12(2):E317–23. doi: 10.1055/a-2226-0840 38420155 PMC10901640

[31] Amato A, Tarantino I, Facciorusso A, Binda C, Crinò SF, Fugazza A, et al. Real-life multicentre study of lumen-apposing metal stent for EUS-guided drainage of pancreatic fluid collections. Gut. 2022;71(6):1050–2. doi: 10.1136/gutjnl-2022-326880 35193984

[32] Maatman TK, Mahajan S, Roch AM, Ceppa EP, House MG, Nakeeb A, et al. Disconnected pancreatic duct syndrome predicts failure of percutaneous therapy in necrotizing pancreatitis. Pancreatology. 2020;20(3):362–8. doi: 10.1016/j.pan.2020.01.014 32029378

[33] Mukai S, Sofuni A, Tsuchiya T, Tanaka R, Tonozuka R, Yamamoto K, et al. A novel puzzle ring replacement technique of a lumen-apposing metal stent with a double-pigtail plastic stent to prevent walled-off necrosis recurrence (with video). DEN Open. 2024;5(1):e70020. doi: 10.1002/deo2.70020 39403557 PMC11472131

[34] Pawa R, Dorrell R, Russell G, Gilliam J, Mishra G, Pawa S. Long-term transmural drainage of pancreatic fluid collections with double pigtail stents following lumen-apposing metal stent placement improves recurrence-free survival in disconnected pancreatic duct syndrome. Dig Endosc. 2022;34(6):1234–41. doi: 10.1111/den.14266 35148447

[35] Bang JY, Mel Wilcox C, Arnoletti JP, Varadarajulu S. Importance of disconnected pancreatic duct syndrome in recurrence of pancreatic fluid collections initially drained using lumen-apposing metal stents. Clinical Gastroenterology and Hepatology: The Official Clinical Practice Journal of the American Gastroenterological Association. 2021;19(6):1275-81.e2. 10.1016/j.cgh.2020.07.022 32683101

